# Newborn Screening for Sickle Cell Disease: Results from a Pilot Study in the Portuguese Population

**DOI:** 10.3390/ijns11010010

**Published:** 2025-01-27

**Authors:** Diogo Rodrigues, Ana Marcão, Lurdes Lopes, Ana Ventura, Teresa Faria, Anabela Ferrão, Carolina Gonçalves, Paula Kjöllerström, Ana Castro, Sofia Fraga, Marta Almeida, Tabita Maia, João Gomes, Ana Lachado, Isabel Guerra, Fátima Ferreira, Fernanda Trigo, Celeste Bento, Laura Vilarinho

**Affiliations:** 1 Newborn Screening, Metabolism and Genetics Unit, Department of Human Genetics, National Institute of Health Doctor Ricardo Jorge, 4000-055 Porto, Portugal; ana.marcao@insa.min-saude.pt (A.M.); lurdes.lopes@insa.min-saude.pt (L.L.); 2 Unidade Local de Saúde de Amadora/Sintra, E.P.E., 2720-276 Amadora, Portugal; ana.ventura@ulsasi.min-saude.pt (A.V.); teresa.m.faria@ulsasi.min-saude.pt (T.F.); 3 Unidade Local de Saúde Santa Maria, E.P.E, 1649-035 Lisboa, Portugal; anabela.ferrao@ulssm.min-saude.pt (A.F.); carolina.a.goncalves@ulssm.min-saude.pt (C.G.); 4 Unidade de Hematologia Pediátrica, Hospital Dona Estefânia, Unidade Local de Saúde São José, 1169-045 Lisboa, Portugal; paula.kjollerstrom@ulssjose.min-saude.pt (P.K.); ana.castro2@ulssjose.min-saude.pt (A.C.); 5 Unidade Local de Saúde de Almada-Seixal, 2805-267 Almada, Portugal; ana.duarte@ulsas.min-saude.pt (S.F.); marta.almeida@ulsas.min-saude.pt (M.A.); 6 Unidade Local de Saúde de Coimbra, 3000-602 Coimbra, Portugal; tabita.maia@ulscoimbra.min-saude.pt (T.M.); 10797@ulscoimbra.min-saude.pt (J.G.); 7 Unidade de Hematologia Pediátrica, Unidade Local de Saúde Santo António, 4099-001 Porto, Portugal; u11598@chporto.min-saude.pt (A.L.); isabelcoutoguerra.pediatria@chporto.min-saude.pt (I.G.); 8 Unidade Local de Saúde São João, 4200-319 Porto, Portugal; mferreira@ulssjoao.min-saude.pt (F.F.); ftrigo@ulssjoao.min-saude.pt (F.T.); 9 Unidade Funcional de Hematologia Molecular, Serviço de Hematologia Clínica da Unidade Local de Saúde de Coimbra, 3000-602 Coimbra, Portugal; celeste.bento@ulscoimbra.min-saude.pt; 10 Associação Portuguesa de Pais e Doentes com Hemoglobinopatias, 2810-274 Almada, Portugal

**Keywords:** newborn screening, sickle cell disease, capillary electrophoresis

## Abstract

The Portuguese Newborn Screening Program currently includes 28 pathologies: congenital hypothyroidism, cystic fibrosis, 24 inborn errors of metabolism, sickle cell disease and spinal muscular atrophy. This pilot study for sickle cell disease newborn screening, including 188,217 samples, was performed between May 2021 and December 2023, with phase I, including 24,130 newborns, in the Lisbon and Setubal districts and phase II, including 164,087 newborns, in the whole country. DBS samples were analyzed through capillary electrophoresis. In phase I, a high birth incidence of sickle cell disease was found (1:928 NBs), resulting from the identification of 24 HbSS and 2 HbSC patients. This birth incidence decreased but remained significant when the pilot study for sickle cell disease newborn screening was expanded to a national level, with the identification of 67 sickle cell disease patients (59 HbSS and 8 HbSC), revealing a birth incidence of 1:2449 NBs. These data suggest that this condition is becoming increasingly relevant in Portugal, thus reflecting a general European trend, where sickle cell disease is already recognized as a public health problem. Therefore, it highlights the importance of its integration into the Portuguese National Newborn Screening Program panel in January 2024, thus allowing the early identification and clinical follow-up of these patients.

## 1. Introduction

The Portuguese Newborn Screening Program started in 1979 and currently includes 28 pathologies: congenital hypothyroidism, cystic fibrosis, 24 inborn errors of metabolism, sickle cell disease and spinal muscular atrophy (pilot study). It is a public health program, carried out voluntarily and covering the whole country, with all samples analyzed in a single national laboratory.

Sickle cell disease (SCD) is one of the most common severe monogenic disorders worldwide. More than 300,000 babies are born with SCD per year, and this number could rise to 400,000 by 2050. The incidence of the disease is high throughout large areas in sub-Saharan Africa, the Mediterranean basin, the Middle East, and India because of the remarkable level of protection that the sickle cell trait provides against severe malaria. SCD has become a public health problem in Europe due to the migratory flows coming from these regions [1,2,3,4].

SCD is an autosomal recessive inherited blood condition presenting with multisystem involvement. The variant responsible for sickle hemoglobin (S hemoglobin) is a single-nucleotide substitution of valine for glutamic acid in the sixth codon of the β-globin chain (HBB:c.20A>T;p.Glu6Val). Common genotypes associated with SCD are homozygous SS disease (HbSS) and the compound heterozygous states HbSC, HbS/β^0^ and HbS/β^+^ thalassemia. Sickle hemoglobin is characterized by reduced solubility, resulting in the development of polymers that damage red blood cells, leading to a decrease in erythrocyte lifespan. Hemolytic and vaso-occlusive phenomena lead to severe clinical complications [5,6,7,8]. The quality and average life expectancy has shown significant improvements due to the prophylactic administration of oral penicillin, anti-pneumococcal vaccination, and parental education regarding the complications associated with the pathology [2,9,10,11]. Prevention of neurological symptoms and timely therapy with hydroxyurea (hydroxycarbamide) have also contributed to improving the prognosis associated with SCD [2,11], although morbidity and mortality are still very high in low-income countries.

This work presents the results of a pilot study for SCD-NBS in 188,217 Portuguese newborns (NBs).

## 2. Materials and Methods

This pilot study was approved by the Ethical Committee for Health from the National Institute of Health Doctor Ricardo Jorge.

Before starting the pilot study for SCD-NBS, and with the support of the Portuguese Association of Parents and Patients with Haemoglobinopathies, an information leaflet was prepared to be provided to the parents at pregnancy surveillance appointments or at the hospital when the baby is born. This leaflet includes information about SCD and the advantages of SCD-NBS, thus allowing an informed decision by the parents, who were informed that they could opt in or out regarding this study with no implications in the other disease NBS and with no need for an additional sample.

The definition of Specialized Treatment Centers for SCD, in the National Health Service, was another essential condition before starting this new screening. Seven centers having specialized pediatric hematology services, and covering all regions of Portugal, were selected for referral of positive cases identified through screening. The collaboration of these centers was requested, including contact and clinical evaluation of referred cases and all necessary steps to confirm the diagnosis, start adequate therapeutic measures, and follow up all newborns.

Dried blood spot (DBS) samples, collected on Guthrie cards, between the 3rd and 6th days of life, were studied in two distinct temporal phases. In phase I, from May 2021 to January 2022, the pilot study for SCD-NBS was conducted at a regional level in the Lisbon and Setubal districts, and 24,130 NB were screened. Phase II started in February 2022, with the expansion to the national level, and until December 2023, 164,087 additional NB were screened. It was also established and transmitted to the sample collection centers that for children submitted to erythrocyte transfusions before collecting the NBS sample, a second sample, collected four months after the last transfusion, was mandatory.

DBS samples were analyzed through capillary electrophoresis using the Sebia Capillarys automated system. The Capillarys 2 Neonat Fast system was used during phase I and the Capillarys 3 DBS system was used during phase II.

All cases with total absence of HbA were reported to specialized treatment centers that are responsible for further elucidation, including distinguishing homozygous sickle (HbSS vs. HbS/β^0^) thalassemia.

DNA extraction and molecular characterization by Sanger sequencing or GAP-PCR was performed for samples with rare hemoglobin variants.

## 3. Results

In phase I, 24,130 NBs from the Lisbon and Setubal districts were screened for SCD. Among these, 26 cases of SCD were identified and reported to specialized treatment centers located in this region: 24 HbSS cases and 2 HbSC cases (Table 1).

In phase II, 164,087 additional NBs were screened. From this cohort, 67 cases for SCD were identified and referred to clinical centers: 59 HbSS cases and 8 HbSC cases (Table 1). In Table 1, we also present the birth incidence of SCD in the Lisbon and Setubal districts (1:928 NBs) and in the whole country (1:2449 NBs). During the pilot study for SCD-NBS, a total of 188,217 newborns were screened. Among these, 93 cases of SCD were diagnosed: 83 HbSS homozygous cases and 10 HbSC compound heterozygous cases.

Furthermore, five cases with other hemoglobin alterations were identified and referred for clinical evaluation, namely four HbEE cases and one β-thalassemia major case. This case was identified due to the total absence of HbA.

There were also detected 3425 carriers of abnormal hemoglobins, of which 626 were detected in phase I and 2799 in phase II. Heterozygotes for abnormal hemoglobin variants were not reported, but it is worth highlighting that the incidence at birth of sickle cell trait in the districts of Lisbon and Setubal (1:45 NBs) is remarkably higher than the one found in the whole country (1:70 NBs) (Table 2).

The cases presenting HbA and an abnormal hemoglobin variant were considered normal carriers of an Hb variant, and a negative result for SCD screening was communicated to the parents. Nevertheless, when an abnormal hemoglobin variant not identified by the Sebia software was detected in a sample also presenting HbA, the sample was anonymized and further studied at the molecular level. The most frequent abnormal hemoglobin variant was Hb Bart’s, caused by homozygous alpha 3.7 deletion, detected by GAP-PCR. For other variants, we performed Sanger sequencing of the HBB, HBA1, and HBA2 genes and detected only variants of the alpha chain, namely Hb J-Paris I (HBA2:c.38C>A;p.Ala13Asp); Hb Oleander (HBA2:c.349G>C;p.Glu117Gln); Hb Chad (HBA1:c.70G>A;p.Glu24Lys) and Hb J-Singa (HBA2:c.235A>G; p.Asn79Asp).

## 4. Discussion

The World Health Organization (WHO) and the United Nations recently identified SCD as a global health burden, and it is largely accepted that optimal care for affected children begins with NBS. In 2016, it was recognized that, due to a large migrant flow from the Middle East and Africa, SCD had become present in all European countries with increasing incidence [12]. SCD has become a public health issue and a challenge for European healthcare systems. This fact prompted a pan-European consensus conference in 2017, resulting in the recommendation for universal SCD-NBS inclusion in all European NBS programs.

England was the first European country to introduce nationwide SCD-NBS (2006) [13], and later, it was extended to the United Kingdom (2014). The Netherlands (2007), Spain (2015), Malta (2017) and Germany (2021) have also included SCD-NBS in their national newborn screening panel. In Belgium, screening is carried out only in the regions of Brussels and Liège. In Italy, universal SCD screening is performed in the Padova-Monza region, while in Novara, Ferrara and Modena, SCD screening is targeted. This is also the case for Ireland and France [3,4,14].

In 1986, a National Program for Haemoglobinopathies Control was implemented in Portugal in collaboration with the WHO, and with the coordination of the Portuguese National Institute of Health Doctor Ricardo Jorge (INSA), a lot of research work was performed in this field. SCD and β-thalassemia major and intermediate are the most common severe forms of hemoglobinopathies in Portugal, and they are more frequent in the center and south of the country. SCD was found to be especially present among communities originating in Africa [10,15,16,17].

To fully understand the Portuguese situation, it is necessary to frame the impact of migratory flows that have historically occurred in Portugal. Throughout our history, several moments have been marked by the influx of immigrants from regions considered at risk for SCD. One example is the large influx of immigrants from Africa in the post-colonial period, who settled in Portugal, mainly in the Lisbon metropolitan area [18,19]. Another relevant moment was Portugal’s integration into the European Union, leading to a new influx of immigrants from African countries, especially from Portuguese-speaking African countries, such as Cape Verde, Angola, and Guinea-Bissau, as well as from other regions of the globe, with a special emphasis on Brazil [19]. More recently, it has been observed that immigrants from Brazil have become the most significant migratory group in the resident population of Portugal. Together with immigrants from Angola, Cape Verde, and Guinea-Bissau, they represent more than half of the resident population with foreign nationality and their presence is no longer restricted to the Lisbon metropolitan area; they are distributed throughout all the national territory [20]. In addition to immigrants from Africa and Latin America, Southwest Asia has also contributed to the growth of the immigrant population in Portugal, consequently leading to the spread of hemoglobinopathies, particularly SCD. The need to identify these patients early is even more pressing, knowing that most of them arise within immigrant communities with low socio-economic conditions.

For this reason, the study was started first in the districts of Lisbon and Setubal, where these communities are largely present, and later extended to the national level.

**Figure 1:**
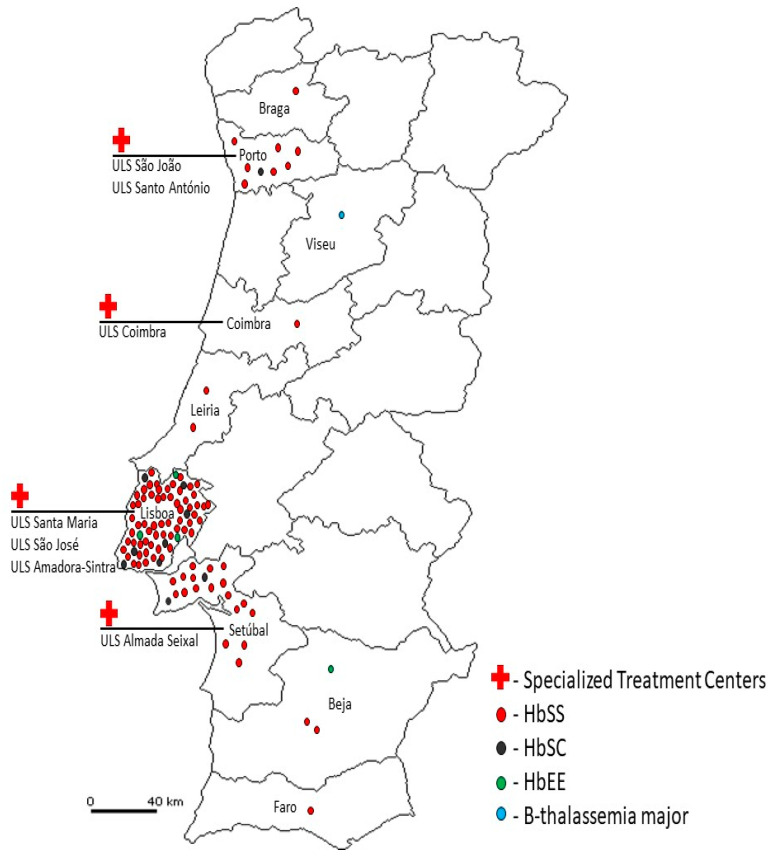
Distribution of patients reported.

This pilot study led to the identification of 93 SCD patients (83 HbSS; 10 HbSC), 4 HbEE homozygous patients and 1 β-thalassemia major patient (Figure 1), who are being properly followed by hematologists in specialized treatment centers.

Additionally, it was possible to identify 3425 carriers of abnormal hemoglobins: 2884 HbAS, 251 HbAC, 168 HbAD and 122 HbAE.

A significant number of transfused babies were identified through the analysis of the hemoglobin profile and further confirmed with the birth hospital, as no information regarding this procedure was recorded on the collection card. We are working closely with health professionals to raise awareness about the importance of indicating transfusion information on the collection card and ensuring compliance with collection requirements. In all such cases, a letter was sent to the parents requesting a recollection at four months of age. Although full compliance with these procedures has not been achieved, no positive screening results have been identified among red-blood-cell-transfused babies. Furthermore, to date, we have no evidence of false-negative results.

The results obtained in phase I confirm a high birth incidence of SCD (1:928 NBs) in the Lisbon and Setubal districts, where 2.2% of the NBs are carriers of hemoglobin S. The results obtained in phase II are more representative of the Portuguese situation, with an SCD birth incidence of 1:2449 NBs and 1.4% hemoglobin S carriers.

As suspected, the contribution of the two districts studied in phase I is significantly higher, confirming that it is the region of the country with the highest birth incidence of SCD. This difference is easily justified by the high number of African-descent immigrants found in these two districts for many years, constituting large communities among which intermarriage is very common and thus contributing even more to the increase in SCD-positive cases. Even if in other regions of Portugal the incidence of SCD is much lower (1:7783), the detection of 13 cases outside of Lisbon and Setubal indicates that the disease is also present there and, for the historical reasons already discussed, it will probably soon become more significant, thus justifying that this screening will continue at a national level.

## 5. Conclusions

In May 2021, the pilot study for SCD-NBS was initiated in Portugal in the districts of Lisbon and Setubal (phase I), revealing a birth incidence for SCD of 1:928 NBs. Subsequently, in February 2022, this pilot study was expanded to include all newborns in Portugal (phase II), revealing a birth incidence of 1:2449 NBs until December 2023. With the results obtained during phase II, we conclude that this condition is increasingly relevant in Portugal, following the European trend where SCD is already recognized as a public health problem. All of this supported the integration of SCD into the panel of the Portuguese Newborn Screening Program in January 2024.

## Figures and Tables

**Table 1:** Cases and birth incidence of SCD in the pilot study for SCD-NBS.

Phase	Region	NB	HbSS	HbSC	Birth Incidence (n)
I—05/2021–01/2022	Lisbon and Setubal	24,130	24	2	1:928 (26)
II—02/2022–12/2023	Portugal	164,087	59	8	1:2449 (67)

**Table 2:** Incidence at birth of carriers of abnormal structural variants of hemoglobin (Hb).

	I—Lisbon-Setubal (*n*)	II—Portugal (*n*)
Carriers of abnormal structural variants of Hb	1:39 (626)	1:59 (2799)
HbAS	1:45 (537)	1:70 (2347)
HbAC	1:561 (43)	1:789 (208)
HbAE	1:1049 (23)	1:1657 (99)
HbAD_G_K	1:1049 (23)	1:1132 (145)

## Data Availability

The data presented in this study are available on request from the corresponding author on reasonable request.
